# HPV-driven oropharyngeal cancer: current knowledge of molecular biology and mechanisms of carcinogenesis

**DOI:** 10.1186/s41199-018-0039-3

**Published:** 2018-12-29

**Authors:** Cassie Pan, Natalia Issaeva, Wendell G. Yarbrough

**Affiliations:** 1 Department of Surgery, Division of Otolaryngology, Yale University, New Haven, CT USA; 2 Department of Otolaryngology/Head and Neck Surgery; Lineberger Cancer Center, University of North Carolina at Chapel Hill, 170 Manning Drive, Campus Box 7070, Chapel Hill, NC 27599 USA

**Keywords:** Oropharyngeal cancer, HPV, Etiology, Treatment

## Abstract

Understanding of oropharyngeal squamous cell carcinoma has significantly progressed over the last decades, and the concept that this disease can be subdivided into two distinct entities based on human papilloma virus (HPV) status has gained acceptance. To combat the constantly growing epidemic of HPV+ oropharyngeal cancer, further investigation and characterization the unique features of the disease, along with the development and implementation of new, targeted therapies, is crucial. In this review, we summarize the etiology, pathogenesis, diagnosis, treatment, and molecular characteristics of HPV-associated oropharyngeal squamous cell carcinoma.

## Background

Head and neck squamous cell carcinomas comprise a diverse group of tumors, which are classified into anatomical subsites including oral cavity, oropharynx, hypopharynx, larynx, and nasopharynx. Cancers of different subsites are known to have unique epidemiology, anatomy, clinical behavior, and association with human papilloma virus (HPV) infection [1, 2]. In this review, we will focus on HPV-driven oropharyngeal squamous cell carcinoma (OPSCC), which has become a matter of growing clinical urgency as its incidence has dramatically increased in recent years. Unique epidemiological, molecular, biological and clinical differences have led to the increasing recognition of HPV-positive OPSCCs as distinct from HPV-negative OPSCCs. This review article will summarize clinical and molecular characteristics of HPV-driven OPSCCs, focusing on factors that distinguish HPV-positive and HPV-negative OPSCCs and examining differences between OPSCC and uterine cervical cancer with attention to an alternative mechanism of HPV carcinogenesis.

### Epidemiology

In the late 20th and early twenty-first century, the campaign to reduce smoking decreased rates of tobacco-related cancers, including oral cavity and laryngeal cancers. During this same period, rates of oropharyngeal cancers increased [3–6]. With the growing number of OPSCCs, the etiologic role of HPV infection also burgeoned, and the percentage of OPSCCs associated with HPV increased from 20% in the 1980s to over 70% by 2005 [7–9]. CDC statistics from 2012 revealed that the incidence of HPV-associated OPSCCs exceeded that of HPV-associated uterine cervical cancers, making OPSCC the most frequently diagnosed cancer caused by HPV [10]. As opposed to HPV-negative cancers of the head and neck, HPV(+) OPSCCs occur in younger patients with minimal or no tobacco exposure [11–16]. HPV(+) OPSCC has a male predominance with men suffering a three to five times higher incidence than women worldwide [16, 17].

Over 90% of HPV(+) OPSCC is caused by the high-risk HPV genotype 16, with almost all oral HPV infections thought to be sexually acquired [14, 18, 19]. The prevalence of oral HPV16 infection in ages 14–69 in the US is ~ 1% (7% for all genotypes), with higher rates in men than in women [19]. The risk for oral HPV infection increases with the number of oral sexual partners, with the higher rates in men being possibly due to men performing oral sex on women and female genitalia carrying a higher HPV burden than male genitalia [17, 20]. Alternatively, since women have a higher seroconversion rate after genital HPV exposure, they may be relatively protected from oral infection [21].

Intense interest regarding the benefits of primary prevention of HPV infection has followed the introduction of HPV vaccines. The Gardasil four-valent vaccine covers HPV types 6, 11, 16, and 18 and received FDA approval for use in females in 2006 and in males in 2011. Since January 2017, the nine-valent Gardasil vaccine with expanded coverage, adding HPV types 31, 33, 45, 52, and 58, has been the only HPV vaccine available in the US. The CDC currently recommends routine vaccination for both girls and boys at age 11–12, with vaccination recommended for females through age 26 and for males through age 21 [22].

A US-based study that examined the effects of HPV vaccination on the burden of oral HPV16 infections found that between 2011 and 2014, vaccination potentially prevented almost one hundred thousand infections [23]. However, due to low vaccine uptake in males, less than half of this effect was seen in men, representing a gap in targeting the most at-risk population [23]. Due to the tepid HPV vaccine uptake and the long latency of developing OPSCC following exposure, it is estimated that the epidemic of HPV(+) OPSCC will continue until 2060 [17].

### Diagnosis

The 2018 version of The National Comprehensive Cancer Network Clinical Practice Guidelines in Oncology (USA) directs that OPSCCs be tested for HPV by p16 immunohistochemistry (IHC) [24]. p16 (p16^INK4a^) IHC has been widely adopted because it is cost effective, reliable, examines paraffin-embedded tissue, and has high sensitivity (94%) [25, 26]. IHC for p16 is particularly good for comparison of HPV(+) and HPV(−) HNSCC, because the protein is overexpressed in HPV(+) HNSCC and frequently lost in HNSCC not associated with HPV [27]. However, in various studies authors have reported that 8–33% of p16-positive OPSCCs lack HPV DNA, likely reflecting a combination of insensitive HPV detection techniques and that p16 overexpression occurs independently of HPV gene expression [28]. To more definitively identify HPV-associated OPSCC, multimodality HPV testing is increasing, with p16 IHC followed by HPV DNA PCR or in-situ hybridization (ISH) being the most common approaches [29]. In the UK, the National Institute for Health and Care Excellence (NICE) recommends reflexing to high-risk HPV DNA or RNA ISH in all p16-positive OPSCCs [30]. Because the specificity of HPV DNA PCR (87%) and ISH (88%) exceed that of p16, the use of these tests in tandem results in increased sensitivity and specificity for HPV detection as opposed to single-modality testing [26]. In addition, HPV DNA testing is being used to diagnose cancer from fine needle aspirates from cervical lymph nodes and to help identify primary tumors [31]. However, the technical challenges and costs of HPV DNA PCR or ISH have limited their use for initial screening.

Next-generation sequencing (NGS) has emerged as an exciting new technology with the potential to identify HPV(+) tumors and provide rich mechanistic and prognostic information distinguishing subsets even within the HPV(+) group. A 2014 study using NGS found that HPV(+) tumors could be further categorized by presence of integrated versus nonintegrated HPV genes and that integration status corresponded with different patterns of DNA methylation and human and viral gene expression profiles in genes with known roles in carcinogenesis [32]. While implications of these findings are unknown, NGS will likely prove clinically useful in the future.

### Prognosis

HPV-positive OPSCC carries a favorable prognosis compared to HPV-negative tumors. Five-year survival rates for patients with advanced stage HPV(+) OPSCC are 75–80%, versus survival rates of less than 50% among patients with similarly staged HPV(−) tumors [33]. The improved survival of patients with HPV(+) tumors can in part be attributed to their remarkable treatment sensitivity, as HPV(+) tumors have been shown to respond better to chemotherapy and radiation than HPV(−) tumors [16, 34]. The better prognosis conferred by HPV positivity is reflected in the updated AJCC 8th edition staging system, which for the first time separates staging for HPV(+) and HPV(−) OPSCCs and in general downgrades HPV(+) OPSCC staging [24, 35]. For example, HPV(+) OPSCC T3 N2, which was classified as Stage IVA in AJCC 7th edition, is newly classified as clinical Stage II in AJCC 8th edition.

Interest in identifying prognostic biomarkers in HPV-associated OPSCC has stemmed from the desire to decrease treatment morbidity while maintaining high cure rates. While a positive p16 by IHC predicts a favorable prognosis regardless of HPV status, recent data has shown that when used together with HPV status, further prognostic stratification is achieved [36]. A 2017 meta-analysis of both OPSCC and HNSCC patients found that the 5-year overall survival was best for patients with HPV(+)/p16(+) tumors, intermediate for HPV(−)/p16(+) tumors, and worst for HPV(+)/p16(−) and HPV(−)/p16(−) tumors [37].

Recent analysis of an HNSCC cohort in The Cancer Genome Atlas (TCGA) identified potential molecular biomarkers that can be used for prognostication [38, 39]. Deletion or mutation of two proteins that inhibit NF-kB and activate interferon, TNF receptor-associated factor 3 (TRAF3) and cylindromatosis (CYLD), were found in 28% of HPV(+) OPSCC [38]. Remarkably, survival for patients with HPV(+) tumors was better for those whose tumors carried defects in either TRAF3 or CYLD, while survival of HPV(+) patients without these mutations was similar to that of HPV(−) negative patients [38].

### Treatment

Despite the prognostic significance of HPV in HNSCC, HPV status has not altered treatment guidelines. For the first time in 2018, The National Comprehensive Cancer Network Clinical Practice Guidelines in Oncology (NCCN, USA), separated treatment pathways for p16(+) and p16(−) OPSCCs [24]; however, recommendations for p16(+) and p16(−) OPSCCs are almost identical, with the only notable difference as follows: as an alternative to definitive radiation therapy (RT) alone or surgery alone, treatment with RT plus systemic therapy is a recommendation (category 2B) for T1 N1 p16-negative tumors, but is not recommended for p16-positive tumors until tumor size reaches T2 (with single node ≤3 cm). In general, regardless of p16 status, RT or surgery remain recommended treatment modalities for early-stage tumors, and combined therapy is recommended for advanced stages. The benefits of induction chemotherapy before concurrent chemoradiation are still being studied, with a recent meta-analysis demonstrating no survival advantage with induction chemotherapy [40].

Standard therapy for advanced OPSCC regardless of HPV status as either definitive or post-operative therapy includes chemotherapy and radiation, which is associated with dose-related adverse side effects, from acute toxicities like mucositis and loss of taste to long-term problems including dysphagia, renal dysfunction, hearing loss, xerostomia, osteoradionecrosis, accelerated arteriosclerosis, neck muscle fibrosis, and trismus. These side effects can lead to a cascade of events, including infections, difficulty eating, and increased hospitalizations, that can markedly erode quality of life. Based on analysis of long-term survivors from the RTOG 91–11 clinical trial, there is also the possibility that treatment-associated morbidity may impact 10-year or longer survival [41]. Given these concerns, minimizing side effects is especially important in advancing therapy for HPV(+) patients, who present at a younger age and have improved survival compared to patients with HPV(−) disease [11–13, 16].

The distinct tumor biology, higher treatment sensitivity, and better prognosis of HPV(+) OPSCCs has piqued interest in therapies that can minimize side effects, including new treatment approaches and de-escalation of current therapies. A single-arm phase II clinical trial, ECOG 1308, examined if response to induction chemotherapy could select stage III-IV (AJCC 7th edition) HPV(+) OPSCC patients for reduced-dose radiation [42]. This trial found that patients with complete response to induction therapy maintained expected tumor control with reduced radiation doses of 54 Gy (compared to 69.3 Gy) but had fewer swallowing problems and nutritional deficiencies. A similar single institution trial also used induction chemotherapy, but stratified HPV(+) OPSCC patients to lower dose radiation (54 Gy) with similar survival and side effect findings [43]. The limited size of both studies as well as the recent changes to the AJCC staging criteria suggest the need for additional larger trials as are currently being considered through the National Clinical Trials Network (NCTN).

Several clinical trials (see Table 1 for currently active or recently completed clinical trials in HPV-associated OPSCC) are examining de-escalated treatments for HPV(+) OPSCCs, include reduced-dose radiation and/or chemotherapy (NCT03215719, NCT03323463, NCT01706939, NCT01898494, NCT02281955, NCT02048020, NCT02215265, NCT02048020), stratifying by responsiveness to induction chemotherapy to select subsequent loco-regional therapy (NCT02281955, NCT03107182), efficacy of chemotherapy or radiation as alternatives to surgery (NCT03210103, NCT03342911), minimally-invasive transoral robotic surgery using pathology to stratify patients for de-escalation (NCT02225496), treatment with surgery alone (NCT02072148), and using targeted therapies (NCT03260023, NCT01855451, NCT02002182, NCT03410615, NCT02540928, NCT03342911).

**Table 1:** displays currently active or recently completed clinical trials in HPV-associated OPSCC (adapted from https://clinicaltrials.gov)

	NCT Number	Title	Interventions
1	NCT03656133	Use of a Proliferation Saturation Index to Determine Personalized Radiotherapy for HPV + Oropharyngeal Cancers	• Radiotherapy fractionation
2	NCT03618134	Stereotactic Body Radiation Therapy and Durvalumab With or Without Tremelimumab Before Surgery in Treating Participants With Human Papillomavirus Positive Oropharyngeal Squamous Cell Caner	• Durvalumab • Modified Radical Neck Dissection • Transoral Robotic Surgery • Tremelimumab
3	NCT03580070	Changes in the Microenvironment of HPV-induced Head and Neck Cancers in West Indies and Metropolitan Population	• Immunotherapy
4	NCT03578406	HPV-E6-Specific TCR-T Cells in the Treatment of HPV-Positive NHSCC or Cervical Cancer	• HPV E6-specific TCR-T cells
5	NCT03418480	HPV Anti-CD40 RNA Vaccine	• HPV vaccine
6	NCT03396718	De-escalation of Adjuvant Radio (Chemo) Therapy for HPV-positive Head-neck Squamous Cell Carcinomas	• De-escalation radio(chemo)therapy - Levels 1 and 2
7	NCT03342911	Nivolumab, Carboplatin, and Paclitaxel in Treating Patients With Stage III-IV Head and Neck Squamous Cell Carcinoma That Can Be Removed by Surgery	• Paclitaxel, Carboplatin, Nivolumab
8	NCT03260023	Phase Ib/II of TG4001 and Avelumab in HPV16 Positive R/M Cancers and Expansion Cohort to Oropharyngeal SCCHN	• TG4001, Avelumab
9	NCT03224000	Trial of Magnetic Resonance Imaging Guided Radiotherapy Dose Adaptation in Human Papilloma Virus Positive Oropharyngeal Cancer	• Modified Barium Swallow, MRI Guided Intensity Modulated Radiotherapy
10	NCT03162224	Safety and Efficacy of MEDI0457 and Durvalumab in Patients With HPV Associated Recurrent/Metastatic Head and Neck Cancer	• MEDI0457, CELLECTRA®5P device, Durvalumab
11	NCT03107182	Chemotherapy and Locoregional Therapy Trial (Surgery or Radiation) for Patients With Head and Neck Cancer	•Carboplatin, Nivolumab, Cisplatin, Hydroxyurea, 5-FU, Dexamethasone, Famotidine, Diphenhydramine, Paclitaxel
12	NCT03077243	P53 Mutational Status and cf HPV DNA for the Management of HPV-associated OPSCC	• Intensity Modulated Radiotherapy, Cisplatin (or alternative)
13	NCT02945631	Quarterback 2 - Sequential Therapy With Reduced Dose Chemoradiotherapy for HPV Oropharynx Cancer	• Radiation: PTV56
14	NCT02865135	Trial To Test Safety And Efficacy Of Vaccination For Incurable HPV 16-Related Oropharyngeal, Cervical And Anal Cancer	• DPX-E7 vaccine
15	NCT02827838	Durvalumab Before Surgery in Treating Patients With Oral Cavity or Oropharynx Cancer	• Durvalumab
16	NCT02784288	Phase II Treatment Stratification Trial Using Neck Dissection-Driven Selection to Improve Quality of Life for Low Risk Patients With HPV + Oropharyngeal Squamous Cell Cancer	•Radiation, Carboplatin, Paclitaxel
17	NCT02706691	BGJ398 in Treating Patients With FGFR Positive Recurrent Head and Neck Cancer	•BGJ398
18	NCT02686008	Pharmacodynamic Study to Assess the Anti-proliferative Activity of the PARP Inhibitor Olaparib in Patients With HPV Positive and HPV Negative HNSCC	• Olaparib
19	NCT02643550	Study of Monalizumab and Cetuximab in Patients With Recurrent or Metastatic Squamous Cell Carcinoma of the Head and Neck	• Monalizumab, Cetuximab
20	NCT02281955	De-intensification of Radiation and Chemotherapy for Low-Risk HPV-related Oropharyngeal SCC: Follow-up Study	• Radiation, cisplatin
21	NCT02215265	Post-operative Adjuvant Treatment for HPV-positive Tumours (PATHOS)	• Cisplatin, Postoperative radiotherapy
22	NCT02178072	Window Trial 5-aza in HNSCC, T-tare	• 5-Azacitadine
23	NCT02113878	Phase Ib Study of BKM120 With Cisplatin and XRT in High Risk Locally Advanced Squamous Cell Cancer of Head and Neck	• BKM120, Cisplatin, Intensity-modulated radiotherapy
24	NCT02002182	ADXS 11-001 Vaccination Prior to Robotic Surgery, HPV-Positive Oropharyngeal Cancer	• ADXS11–001 (ADXS-HPV)
25	NCT01716195	Induction Chemotherapy Followed by Chemoradiotherapy for Head and Neck Cancer	• Radiotherapy
26	NCT01706939	The Quarterback Trial: Reduced Dose Radiotherapy for HPV+ Oropharynx Cancer	• Reduced Dose Radiation, Carboplatin
27	NCT01530997	De-intensification of Radiation & Chemotherapy in Low-Risk Human Papillomavirus-related Oropharyngeal Squamous Cell Ca	• Intensity Modulated Radiotherapy, Cisplatin
28	NCT01302834	Radiation Therapy With Cisplatin or Cetuximab in Treating Patients With Oropharyngeal Cancer	• cetuximab, cisplatin

In addition to de-escalation of standard therapy, new treatment modalities for HPV+ HNSCC are being developed with the hope of decreasing morbidity of current therapies. Early promising results from an ongoing clinical trial published in 2017 from the Yale Cancer Center examined molecular effects of DNA-demethylation using 5-azacytidine (5-azaC) for treatment of HPV(+) HNSCC patients [44], Table 1. Preclinical data revealed that 5-azaC inhibits growth and increases cell death of HPV(+) cancer cells associated with reduced expression of HPV genes, stabilization of p53, and activation of p53-dependent apoptosis. Evaluation of HPV(+) OPSCC tumor specimens from trial patients treated with 5-azaC (75 mg/m^2^ for 5–7 days) reinforced the pre-clinical data, showing increased p53 expression, increased apoptosis and decreased expression of HPV genes. In a mouse xenograft model, 5-aza was also found to reduce the metastatic potential of HPV(+) tumors. A larger clinical trial is needed to fully characterize the therapeutic potential and safety of this promising therapy in HPV(+) HNSCC.

One mechanism of immune escape in HNSCCs is mediated by the receptor programmed death − 1 (PD-1) interacting with its ligand, PD-L1, which is expressed in 50–60% of HNSCC and 70% of HPV(+) HNSCCs [45]. PD-L1, found to be selectively expressed in tonsillar crypts, may facilitate HPV infection at these sites, reflecting the potential for targeted therapy with PD-1 inhibitors in HPV(+) cancers [46]. Two PD-1 inhibitors, pembrolizumab (KEYTRUDA, Merck Sharp & Dohme) and nivolumab (OPDIVO, Bristol-Myers Squibb), were approved by the FDA for the treatment of recurrent or metastatic HNSCC that failed a platinum-based therapy [47, 48]. Studies examining the efficacy of these drugs in patients with recurrent or metastatic HNSCC revealed a relatively low overall response rate of 13–18% with no difference in response between HPV(+) and HPV(−) cancers [45]. Many clinical trials examining combinations of immune checkpoint inhibitors with other immune modulators, radiation, cytotoxic chemotherapy or epigenetic therapies are underway [49].

### Molecular characteristics

In addition to the epidemiological, pathological, and clinical characteristics distinguishing HPV(+) and HPV(−) OPSCCs, TCGA and other efforts have elucidated molecular and epigenetic differences [50–52]. Here, we will explore heterogeneity between HPV(+) and HPV(−) HNSCC, as well as distinctions within OPSCC driven by HPV with particular attention on defects that correlate with tumor response and patient survival.

Altered DNA-repair pathways, differences in mitogenic signaling pathways, dysregulation of cell cycle control, and changes in the tumor micro-environment of HPV(+) tumors have all been proposed as possible explanations of their enhanced sensitivity to radiation [53]. SMG-1 (suppressor with morphogenetic effect on genitalia) is a member of the phosphoinositide 3-kinase-related kinases (PIKK) family and plays an important role in the DNA-damage response [54–56]. In OPSCCs, expression of SMG-1 was found to be decreased in HPV(+) tumors due to hypermethylation of its promotor [57]. This decreased expression of SMG-1 appears to be an important contributor to the radiosensitivity of HPV(+) cells, as depletion of SMG-1 in HPV(−) cells was shown to cause increased radiosensitivity while overexpression in HPV(+) protected cells from radiation [57]. Further evidence of the altered mechanisms in DNA repair in HPV(+) tumors was seen through reverse-phase protein array (RPPA) profiling of OPSCCs, which found that all eleven DNA repair proteins screened, including BRCA2, MSH2, PARP-1, and ATM, were significantly upregulated in HPV(+) samples compared to HPV(−) samples [58]. This is particularly interesting, since HPV(+) oropharyngeal cancer cells have shown a partial deficiency in DNA double strand breaks repair mostly in the homologous recombination repair pathway [59, 60] that may also contribute to increased sensitivity to radiation or DNA damaging agents.

Differences in cell cycle regulation may also play a role in the remarkable treatment sensitivity of HPV(+) tumors. Amplification and overexpression of cyclin D1, inactivation of the cyclin-dependent kinase inhibitor p16, and mutations of the tumor suppressor p53 are common defects found in HPV(−) HNSCC, but are lacking in HPV(+) tumors [50, 61]. On the other hand, amplification and overexpression of E2F1, which is a driver of G1-to-S transition, is common in HPV(+) but not HPV(−) HNSCC [50, 61]. Given these differences in cell cycle regulation, it is not surprising that differences to treatment with cyclin-dependent kinase (CDK) inhibitors are observed. HPV-positivity has been shown to correlate with hypersensitivity of tumor cells to roscovitine, a cyclin-dependent kinase (CDK) inhibitor that inhibits CDK-1, 3, 5, 7, and 9 [62]. Treatment of HPV(+) OPSCC cells with roscovitine resulted in DNA-damage and induced a p53-dependent cell death [62]. Additionally, low-doses of roscovitine that did not cause weight loss in mice significantly inhibited growth of HPV(+) xenografted tumors.

In addition to differences distinguishing HPV(+) and HPV(−) head and neck tumors, significant molecular heterogeneity exists within HPV(+) tumors themselves. Gene expression profiling has classified HPV(+) tumors into two subgroups with one (HPV-KRT) having elevated expression of genes involved in keratinization, viral integrations, spliced E6, chr3q amplifications, and PIK3CA mutations, and the other subgroup (HPV-IMU) having more mesenchymal differentiation, full-length E6 activity, chr16q deletions, and a stronger immune response [63]. Although TCGA survival analysis showed a trend toward better survival in the HPV-IMU subgroup, the survival difference was not significant, warranting further investigation into the clinical implications of this HPV(+) stratification model [63].

Further proving the heterogeneity of HPV(+) HNSCCs, a 2014 study of HNSCCs from TCGA found that of the 35 HPV(+) tumors, 25 had integration of the viral genome while 10 tumors lacked integration [32]. The canonical paradigm of HPV carcinogenesis, which was developed through studies of uterine cervical cancer, highlights the importance of HPV genome integration as premalignant lesions transition to become malignant [64]. Discovering that nearly 30% of HPV(+) OPSCC contained only episomal HPV challenged this canonical theory of HPV carcinogenesis and presented an opportunity for understanding alternative mechanisms of HPV-driven tumorigenic conversion.

OPSCC with integrated versus nonintegrated HPV have differences in somatic gene methylation, gene expression patterns, mRNA processing, and inter- and intrachromosomal rearrangements [32]. For tumors with HPV integration, integration was not random, with many integration sites occurring within cancer-associated genes suggesting that even within tumors with integrated HPV, different molecular events may be involved in carcinogenesis [32, 65]. Given the molecular differences that are based on HPV integration status, it is not surprising that clinical parameters also differ. In support of this, absence of integration correlated with improved survival and with indications of increased immune infiltration [65]. Recently, defects in TRAF3 and CYLD were found as novel alterations in HNSCC that identified a subset of HPV(+) HNSCC with improved survival [38, 50]. TRAF3 and CYLD gene deletions or disruptive mutations were identified in 28% of HPV(+) specimens in the initial TCGA HNSCC cohort and correlated with the absence of HPV gene integration [38]. Consistent with known functions of TRAF3 and CYLD, tumors with altered TRAF3 or CYLD had activation of NF-kB and inactivation of innate immune signaling [38, 39]. These gene defects were nearly significant in correlating with decreased tobacco exposure in this cohort, raising the possibility that DNA damage, reactive oxygen species or other factors associated with tobacco smoke may increase the probability of HPV integration. In light of finding that TRAF3 or CYLD mutation or deletion identified a unique subset of HPV(+) patients, additional analysis of genes regulating the NF-kB pathway were examined in an independent Yale cohort (unpublished data). This analysis confirmed the existence of TRAF3 and CYLD mutations, but also found defects in additional NF-kB regulators (MAP3K14, BIRC3, TRAF2, and MYD88). This cohort is being followed, but time from treatment for this cohort is currently too short to draw survival conclusions. Identification of additional mutations in regulators of NF-kB suggest that NF-kB pathway defects in addition to TRAF3 and CYLD may be important for separating subtypes of HPV(+) OPSCC tumors.

### Etiology

The mechanisms of HPV-driven OPSCC have not been intensely studied, as many have assumed that HPV carcinogenesis in OPSCC is identical to the accepted mechanism of HPV carcinogenesis in the uterine cervix; however, there are many differences between HPV(+) OPSCC and uterine cervical cancer. HPV(+) OPSCC and cervical cancer diverge in epidemiologic factors, molecular patterns, HPV type, mutational profile, cell-of-origin, treatment response, and clinical behavior (Table 2), suggesting that uterine cervical cancer and OPSCC are distinct [66]. While more than 85% of cervical cancer cases worldwide are from developing nations, the developing world has relatively fewer OPSCCs than higher-income countries [66]. Furthermore, over 90% of HPV(+) OPSCCs are caused by HPV16, whereas in cervical cancer, only 50% is attributable to HPV16 and up to 20% is caused by HPV18, which is rarely identified in OPSCC [14, 67]. HPV(+) OPSCC and uterine cervical cancer mutational landscapes also differ; for example, in an analysis of an expanded TCGA cohort, almost 30% of HPV(+) oropharyngeal tumors had TRAF3 or CYLD mutations or deletions, while these mutations were extremely rare in cervical cancer [38]. From a clinical standpoint, HPV(+) OPSCCs respond better to treatment than HPV(+) cervical cancer, possibly due to differences in the unique properties of their respective epithelial sites of infection, clinical presenting signs and symptoms, patterns of metastasis, and target populations, but also possibly due to molecular differences [66]. Clinical and molecular differences between OPSCC and uterine cervical cancer should caution against equating any aspect of these HPV-associated diseases including carcinogenesis, treatment response or outcome.

**Table 2:** Major differences between cervical cancer and HPV-associated OPSCC

	OPSCC	Cervical Cancer
Incidence	Incidence increasing	Incidence decreasing
Prevalence	Increased in higher-income countries	Increased in lower-income countries
Sex	> 70% male	100% female
Etiology	Tobacco and alcohol remain important causes, along with HPV	Virtually all are caused by HPV
HPV genotype	> 95% HPV16 HPV18 rare	50% HPV16 20% HPV18
Premalignant lesions	Unknown	CIN1–3
Screening tests available	No	Yes
5-year survival rate	> 75%	< 70%
TRAF3/CYLD mutations	Approximately 30%	Rare
Treatment sensitivity to chemotherapy and radiation	High	Moderate

The productive HPV life cycle has been studied and is outlined here [68]. In the uterine cervix, HPV gains access through microabrasions to infect basal epithelial cells, and after infection, the HPV genome replicates to a low copy number to be maintained as nuclear episomes. HPV early genes are expressed at low levels, and after the initial low-level amplification, HPV episomes are maintained through replication in sync with cell division. These characteristics are thought to assist with immune evasion by minimizing activation of pattern recognition receptors, NF-kB, and downstream type I interferon signaling. Emphasizing the importance of immune system evasion in HPV life cycle, proteins encoded by HPV inhibit NF-kB and type I interferon signaling [69]. Cellular differentiation is critical for the final productive amplification stage of the HPV life cycle. As cells migrate toward the surface of the epithelium, differentiation triggers increased expression of E6 and E7 oncoproteins that in turn enables an expansion of DNA replication-competent cells and a several log amplification of HPV episomes, ultimately concluding with expression of late viral capsid genes, encapsidation of HPV genomes, and shedding of new viral particles from the epithelial surface [70, 71]. Most mucosal infections are cleared within two years through activation of innate and acquired immune mechanisms [72, 73]. The importance of acquired immunity in HPV clearance is supported by identification of HLA variants associated with decreased risk of both HPV(+) OPSCC and uterine cervical cancer [74]. On the other hand, persistent infection predisposes to malignant transformation that requires additional mutations and/or immune deficiency. The delay between HPV infection and detection of malignancy can be several decades [17, 70].

Studies of the transformation process initiated by HPV infection have relied heavily on the study of premalignant uterine cervical cells and have led to a canonical model of HPV carcinogenesis. In this model, initial infection, establishment and maintenance are thought to parallel the normal HPV life cycle; however, with persistent infection of basal or stem cells carcinogenesis can be initiated. The model details that as cells progress from early dysplasia (CIN1) to pre-malignant lesions (CIN3), the HPV genome integrates and disrupts the HPV E2 gene, which relieves negative feedback and increases expression of HPV oncoproteins, E6 and E7 [72, 75–77]. As opposed to the natural life cycle where E6 and E7 expression increases in the superficial layers of the epithelia, the carcinogenesis model establishes cells with high E6 and E7 expression at the basal layer of the epithelium where in the absence of immune clearance, these pre-malignant cells persist. Increased expression of HPV oncoproteins inactivates the major human tumor suppressor genes, p53 and RB leading to genomic instability, resistance to apoptosis, and dysregulated cell cycle control. One caveat of integration studies that contributed to the model is that methods for identification of integrated HPV frequently relied on loss of E2, and by design, these techniques exclude integrated forms that maintain E2 [77]. While integration of the HPV genome is part of the canonical HPV carcinogenesis model, it excludes a percentage of HPV type 16-positive cervical cancers that lack detected HPV integration [77]. It is unclear if this model applies to a portion of OPSCC with integration, but it is evident that it does not describe carcinogenesis for HPV(+) OPSCC that lack integration of the HPV genome.

TCGA analysis of HPV-associated OPSCC provides some characterization of the role of HPV integration in tumors [32]. Genomic profiling revealed that HPV-driven carcinogenesis is more complex and heterogeneous than previously thought. In OPSCC, HPV integration was associated with breakpoints throughout the viral genome, with only breakpoints in E1 occurring more frequently than expected by chance. This finding contrasts with the canonical HPV carcinogenesis model, in which disruption of E2 through integration leads to enhanced expression of E6 and E7 [72]. Further complicating the picture, whole genome sequencing data identified a category of tumors containing both partially deleted HPV genomes and full-length genomes [78]. The status of HPV in these “mixed” tumors remains controversial, with some authors describing these tumors as containing both integrated and nonintegrated HPV, while others argue that these tumors represent viral-human hybrid episomes [79].

Direct analysis of the HPV carcinogenesis process in the tonsil is not possible due to the absence of a defined pre-malignant lesion. The area infected by HPV and prone to transformation within the tonsil – the tonsillar crypt – lacks tight epithelial junctions and is characterized by incomplete basement membranes making pathological distinction between invasive cancer and intra-epithelial neoplasia impossible [80, 81]. In fact, the College of American Pathologists 2017 Guidelines state that in-situ disease in HPV(+) oropharyngeal cancer is “non-existent” [82]. In addition, murine modeling of OPSCC may not recreate the human situation because mice do not have tonsils and therefore lack the tonsillar crypt cells that are the target of carcinogenic HPV infection.

Given the difficulties of studying progression of HPV pre-malignancies in the oropharynx, comparison of molecular characteristics of OPSCC and uterine cervical cancer may shed light on mechanisms of carcinogenesis in these distinct cancers. APOBEC (apolipoprotein B mRNA editing enzyme, catalytic polypeptide-like) is a cytidine deaminase whose mutations have been implicated in carcinogenesis. Both OPSCC and uterine cervical cancers have enrichment for APOBEC mutational signatures [50, 83, 84]. Consistent with finding APOBEC mutational signatures in each, both tumor types have a significant burden of APOBEC-driven PIK3CA mutations, and activating mutations of PI3KCA occur more frequently in HPV(+) than HPV(−) OPSCCs [50, 58, 84]. Enhanced PI3K signaling has been implicated in HNSCC tumorigenesis; however, the role of PI3K pathway activation by PIK3CA mutations in HPV(+) OPSCC needs to be further explored since AKT was not activated by mutant PIK3CA in the presence of HPV oncoproteins [58, 85, 86]. Cervical cancers and OPSCCs also share an absence of mutations in genes or pathways regulated by HPV oncoproteins such as p53 and p16^INK4a^, confirming the importance of HPV oncogenes in tumorigenesis [50, 84]. Interestingly, EGFR (17%) and ERB2 (17%) amplifications were found in uterine cervical cancer, but not in HPV(+) OPSCC, while FGFR3 amplifications were found in 11% of HPV(+) OPSCC, but not in uterine cervical cancer, suggesting that these tumors rely differently on receptor tyrosine kinase signaling [50, 84].

For tumors caused by HPV type 16, integration of the HPV genome occurs at similar rates in OPSCC (72%) and cervical cancer (76%) [32, 84]. Despite the similar proportion of tumors lacking integration, the strong correlation of TRAF3 and CYLD defects with the absence of integration in OPSCC compared to the lack of these defects in uterine cervical cancer suggest that HPV carcinogenesis in tumors lacking integration may differ [38, 50, 84]. Although the reason for this difference is unknown, the function of TRAF3 and CYLD as inhibitors of NF-kB and activators of type I interferon signaling suggest that disruption of these genes may be critical for survival of infected cells and maintenance of unintegrated HPV DNA in oropharyngeal cells. A recent study confirmed that attenuated TRAF3 activated NF-kB and inhibited interferon in HPV(+) HNSCC cells [87]. The reason that TRAF3 or CYLD mutations are not required in uterine cervical cells is unknown but could relate to the differences in the infected cell. Unlike uterine cervical cells at the squamocolumnar junction, tonsillar crypt cells are closely associated with non-epithelial and lymphatic cells [88]. The lymphoepithelium of the tonsil is critical for initiation of immune responses with one role of the specialized crypt epithelial cells being endocytosis to deliver antigens to adjacent immune cells that initiate immune responses through antigen processing and release of cytokines [88]. Several pathogens take advantage of the discontinuous epithelium and immune milieu to invade, including the Epstein-Barr Virus (EBV), which infects the lymphoepithelium of the nasopharynx and can result in nasopharyngeal cancer [88]. Like HPV, EBV infects many years before cancers develops and must be maintained for carcinogenic conversion. Unlike HPV, EBV is a herpesvirus, which does not integrate and therefore must be maintained in an episomal form [89]. Interestingly, EBV-associated nasopharyngeal cancer is one of the few solid tumor types other than HPV(+) OPSCC that has TRAF3 mutations [38, 90]. Inhibition of NF-kB signaling in EBV-associated nasopharyngeal cancer cells has been shown to inhibit their growth, suggesting that the cells are reliant on continuous NF-kB activity [90].

Together these data raise the intriguing possibility of an alternative mechanism of HPV carcinogenesis uncovered through the study of HPV(+) OPSCC. Instead of HPV integration as a driver for increased oncoprotein expression, a subset of OPSCC may rely on maintenance of unintegrated HPV that in turn requires molecular defects in TRAF3, CYLD or other genes to activate NF-kB and inhibit innate immune responses.

## Conclusions

Recent studies of OPSCC are increasing our understanding of HPV-associated carcinogenesis, including the possibility of an alternative mechanism reliant on activation of NF-kB, inhibition of interferon and maintenance of non-integrated HPV. In addition, markers to identify HPV(+) OPSCC patients with improved prognosis are emerging. These insights are critical to improving our management of this rising disease and exploring effective new treatments and identification of patients for de-escalated therapy.

## Abbreviations

5-azaC: 5-azacytidine
APOBEC: Apolipoprotein B mRNA editing enzyme, catalytic polypeptide-like
CDK: Cyclin-dependent kinase
CYLD: Cylindromatosis
EBV: Epstein-Barr Virus
HNSCC: Head and neck squamous cell carcinoma
HPV: Human papillomavirus
ISH: In-situ hybridization
NICE: National Institute for Health and Care Excellence
OPSCC: Oropharyngeal squamous cell carcinoma
PD-1: Programmed death − 1
PIKK: Phosphoinositide 3-kinase-related kinases
RPPA: Reverse-phase protein array
RT: Radiation therapy
SMG-1: Suppressor with morphogenetic effect on genitalia
TRAF3: TNF receptor-associated factor 3
